# Fetal MRI Characteristics of Exencephaly: A Case Report and Literature Review

**DOI:** 10.1155/2016/9801267

**Published:** 2016-02-03

**Authors:** Ali Sharif, Yihua Zhou

**Affiliations:** Department of Radiology, Saint Louis University School of Medicine, 3635 Vista Boulevard at Grand Boulevard, Saint Louis, MO 63110, USA

## Abstract

We present the fetal MRI characteristics of exencephaly, a rare malformation of the cranium. The fetus was initially misdiagnosed as anencephaly at 14 weeks of estimated gestational age (EGA) and later mislabeled as acrania at 20 weeks of EGA by ultrasound. A confirmatory magnetic resonance imaging (MRI) at 29 weeks of EGA demonstrated findings consistent with exencephaly, which was confirmed after birth. To our knowledge, no full fetal MRI characteristics have been described. We hope to use this case to review the key MRI findings in differentiating exencephaly from other cranial vault defects and to help early diagnosis of exencephaly as the appropriate use of correct nomenclature allows better research while giving parents the most accurate and appropriate counseling.

## 1. Introduction

Exencephaly is a rare malformation of the cranium that is characterized by the absence of the skull, the cranial cavity, and the scalp with a large mass of protruding brain tissue covered by a membrane and with prominent bulging eyeballs [1]. It is considered to be an embryological precursor of anencephaly. In exencephaly, there is only a vascular layer of epithelium covering the brain tissue, which is slowly degraded during gestation by the amniotic fluid and degenerates into anencephaly [2]. Due to this pattern of progression, anencephaly is considered relatively more common than exencephaly [3]. In this paper, we report the prenatal magnetic resonance imaging (MRI) features of exencephaly that was confirmed in a live birth and review the MRI findings of anencephaly and other congenital cranial vault defects including acrania and acalvaria.

The MRI imaging features of cranial vault defects have been described in postnatal live births and postmortem fetuses [1]. However, for prenatal diagnosis, ultrasound remains the primary modality. To our knowledge, no full fetal MRI characteristics have been described. We hope to use this case to review the key MRI findings differentiating exencephaly from other cranial vault defects and to help early diagnosis of exencephaly as the appropriate use of correct nomenclature allows better research while giving the parents the most accurate and appropriate counseling [4].

## 2. Case Presentation

A 28-year-old female (gravida 2, para 2) was transferred from an outside facility for ultrasonographic features concerning anencephaly. The fetus was initially diagnosed as anencephaly at approximately 14 weeks of estimated gestational age (EGA). A subsequent ultrasonographic evaluation suggested a diagnosis of acrania at approximately 20 weeks of EGA.

**Figure 1:**
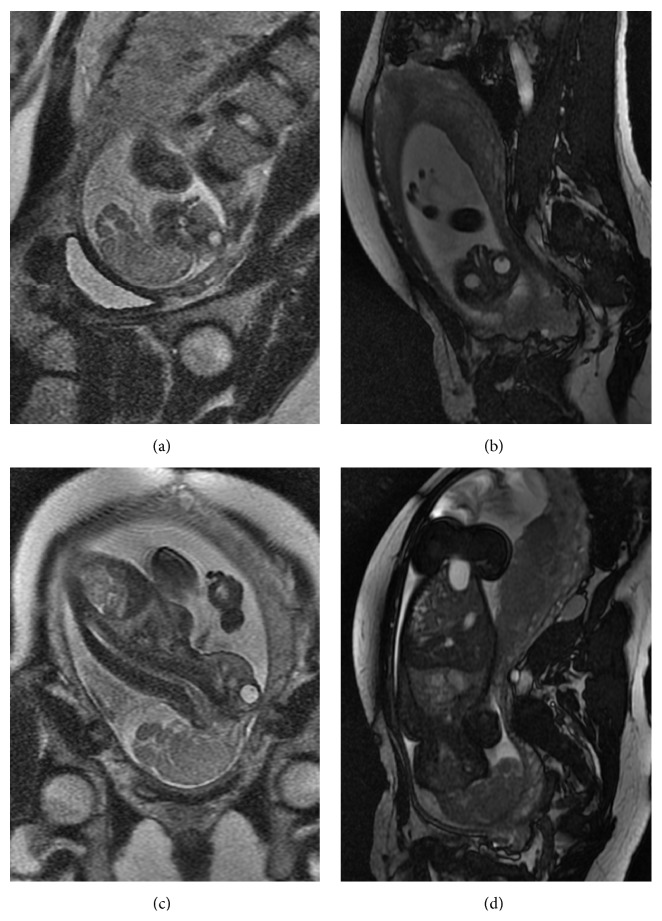
(a) Sagittal view fetal MRI demonstrating the absence of the calvarium with large mass of brain tissues hanging outside of the skull base and an incomplete layer of vascular epithelium covering the deformed brain. (b) Coronal view fetal MRI showing the intact skull base with normal appearing orbits and nasal cavity. (c) Sagittal view fetal MRI depicting the absence of the calvarium but intact orbits and skull base. The spine is normal without evidence of myelomeningocele. (d) Coronal view fetal MRI demonstrating exencephaly with grossly intact body organs.

A confirmatory MRI performed at our fetal care institute at 29 weeks of EGA demonstrated the absence of the fetal calvarium, missing the frontal, parietal, occipital, and temporal bones as well as the scalp. The brain tissues including the brain stem and cerebellum were enlarged, deformed, and protruded out through a defect above the skull base (Figure 1(a)). There was an incomplete membranous structure covering the brain tissue (Figure 1(a)). The ventricular system was deformed with loss of normal configuration. The skull base and the face had developed normally with normal appearing eyes, nose, and mouth (Figure 1(b)). The fetal spine and spinal cord appeared normal. There was no evidence of myelomeningocele or other types of spinal dysraphism or segmental abnormalities (Figures 1(c) and 1(d)). These features were consistent with exencephaly.

The fetus was at breech presentation at term and consequently required a cesarean section to deliver a live male baby. At birth, the calvarium was absent and the exposed neural tissue was visible. The brain tissue was covered with a thin layer of friable membrane and it was subsequently covered with surgical dressings. The baby was bradycardic and hypoxemic at birth and was consequently resuscitated. The facial features were normal. However, the tongue was noted to be retropositioned, requiring oral airway support. The APGAR scores were 2, 5, and 8, at 1, 5, and 10 minutes, respectively. The baby survived for approximately 5 hours after birth.

## 3. Discussion

Congenital cranial vault defects refer to conditions with similarly dismal prognoses, including exencephaly, anencephaly, acalvaria, and acrania [5]. There are confusions regarding the use of these terminologies. Even in the literature, there are contradictory descriptions of these conditions. In this case report, there was clear demonstration of the confusion in choosing the correct diagnosis, as it was first called anencephaly at 14 weeks of EGA and later mislabeled as acrania at 20 weeks of EGA at fetal ultrasound examinations.

Fetal MRI has increasingly become a tool for confirmation of ultrasound findings. We believe that the MRI features described in this report can help early diagnosis of the condition. The appropriate use of correct nomenclature allows better research while giving parents the most accurate and appropriate counseling [4].

Table 1 summarizes the differences among the four entities of cranial defect. Exencephaly is an uncommon malformation of the cranium, with a characteristically large disorganized mass of brain tissue [6]. It is considered to be an embryological precursor of anencephaly. In exencephaly, there is only a vascular layer of epithelium covering the brain tissue, which is slowly degraded during gestation by the amniotic fluid and the brain degenerates into anencephaly [2]. The characteristic fetal MRI for exencephaly include

- absent calvarium and scalp above the orbits,
- intact skull and normal development of the face including the eyes, nose, and mouth,
- distortions of the brain parenchyma with loss of landmarks,
- the brain tissue being covered by an incomplete membranous structure,
- deformity of the cerebellum and brain stem.

Anencephaly, which is thought to be relatively more common than exencephaly [3], is the result of a sequence of destruction of exposed neural tissue resulting in absent cerebral hemispheres. The development of the forebrain is disrupted and all that remains is the area cerebrovasculosa with a flattened remnant of disorganized brain tissue admixed with ependymal, choroid plexus, and meningothelial cells [7]. The cranial defect is only covered by angiomatous stroma (area cerebrovasculosa).

Acalvaria is also characterized by absent membranous calvarium. However, there is an intact skin covering the extracranial brain tissues. The cerebral hemispheres, skull base, and facial bones are also preserved. The dura is intact and complete [8], unlike exencephaly.

Acrania is reserved for cases where the entire neurocranium (including the skull and skull base) is absent with complete but abnormal development of brain tissue. The cerebral hemispheres are surrounded by a thin membrane [2].

In conclusion, due to their poor prognosis, it is important to correctly diagnose and distinguish different forms of congenital cranial defects, including exencephaly, anencephaly, acalvaria, and acrania, particularly in the early stage of fetal development. As shown in this case, appropriate fetal MRI imaging can provide confirmatory evidence for diagnosis of exencephaly when fetal ultrasound is equivocal. Fetal MRI findings of exencephaly include absence of the scalp and skull above the orbits but with normally developed skull base and face as well as malformed cerebral hemispheres and ventricles.

## Figures and Tables

**Table 1:** Comparison of the four cranial vault defect entities.

	Exencephaly	Anencephaly	Acalvaria	Acrania
Cerebrum	Enlarged, disorganized, and deformed mass of brain tissue	Flattened remnant of disorganized forebrain tissue admixed with ependymal, choroid plexus, and meningothelial cells	Present but deformed	Present but deformed

Cerebral hemispheres	Present but deformed	Absent	Present	Present

Cerebellum	Present but deformed	Absent	Present	Present

Covering of extracranial brain tissue	Vascular layer of epithelium	Angiomatous stroma (area cerebrovasculosa)	Dura and skin	Thick membrane

Calvarium	Absent above the orbits	Absent above the orbits	Absent above the orbits	Absent completely

Skull base	Normal	Normal	Normal	Absent

Facial structures	Normal	Deformed	Normal	Normal
